# Correction: Does menopause influence the association between atherogenic index of plasma and prediabetes? A cross-sectional study in middle-aged Chinese women

**DOI:** 10.1371/journal.pone.0348212

**Published:** 2026-04-27

**Authors:** The PLOS One Staff

The authors Bin Ouyang, Hongxia Zhuo and Huiwu Han should not have been attributed equal contribution to this work, only Hongxia Zhuo and Huiwu Han.

The publisher apologizes for the error.
