# Supplementary material for: Does menopause influence the association between atherogenic index of plasma and prediabetes? A cross-sectional study in middle-aged Chinese women
Source: PLoS One. 2026 Feb 12;21(2):e0342644. doi: 10.1371/journal.pone.0342644 (PMC12900311; doi:10.1371/journal.pone.0342644)
Supplement: S2 Appendix — (DOCX) [file pone.0342644.s002.docx]

**S2 Appendix**

**Table S1** The proportion of missing variables (n=12,885).

| **Variables** | **Values** | **proportions (%)** |
| --- | --- | --- |
| Age | 0 | 0.00 |
| Marital status | 285 | 2.21 |
| Education | 870 | 6.75 |
| Occupation | 261 | 2.03 |
| Family history of diabetes | 268 | 2.08 |
| Age of menarche | 263 | 2.04 |
| Menopausal status | 290 | 2.25 |
| Age at first childbirth | 351 | 2.72 |
| Breastfeeding time | 603 | 4.68 |
| Gestational diabetes | 603 | 4.68 |
| Gestational hypertension | 603 | 4.68 |
| Smoking status | 261 | 2.03 |
| Drinking status | 261 | 2.03 |
| Exercise or not | 261 | 2.03 |
| BMI | 630 | 4.89 |
| WC | 245 | 1.90 |
| HC | 246 | 1.91 |
| SBP | 202 | 1.57 |
| DBP | 202 | 1.57 |
| FPG | 24 | 0.19 |
| HbA1c | 7123 | 55.28 |
| OGTT 2h-PG | 12833 | 99.60 |
| TC | 19 | 0.15 |
| TG | 19 | 0.15 |
| HDL-C | 20 | 0.16 |
| LDL-C | 20 | 0.16 |
| ALT | 23 | 0.18 |
| AST | 7368 | 57.18 |
| BUN | 23 | 0.18 |
| Scr | 246 | 1.91 |
| UA | 23 | 0.18 |

AIP: atherogenic index of plasma, ALT: alanine aminotransferase, BMI: body mass index, BUN: blood urea nitrogen, DBP: diastolic blood pressure, FPG: fasting plasma glucose, HbA1c: glycosylated hemoglobin, HC: hip circumference, HDL-C: high-density lipoprotein cholesterol, LDL-C: low-density lipoprotein cholesterol, OGTT:oral glucose tolerance test, SBP: systolic blood pressure, Scr: serum creatinine, TC: total cholesterol, TG: triglycerides, UA: uric acid, WC: waist circumference, 2h-PG:2-hour Plasma Glucose
