# Supplementary material for: Does menopause influence the association between atherogenic index of plasma and prediabetes? A cross-sectional study in middle-aged Chinese women
Source: PLoS One. 2026 Feb 12;21(2):e0342644. doi: 10.1371/journal.pone.0342644 (PMC12900311; doi:10.1371/journal.pone.0342644)
Supplement: S3 Appendix — (DOCX) [file pone.0342644.s003.docx]

**S3 Appendix**

**Table S2** Baseline characteristics of participants classified by quartiles of AIP (n=7,929).

| **Variables** | **AIP-quartile** | | | | ***P*** |
| --- | --- | --- | --- | --- | --- |
|  | **Q1(n=2,120)** | **Q2(n=2,002)** | **Q3(n=1,964)** | **Q4(n=1,843)** |  |
| **Age (years)** | 51.00(48.00,54.00) | 51.00(48.00,54.00) | 52.00(49.00,55.00) | 52.00(49.00,55.00) | <0.001 |
| **Marital status** |  |  |  |  | 0.469 |
| Married | 2023(95.42) | 1897(94.76) | 1864(94.91) | 1769(95.98) |  |
| Divorced or widowed | 81(3.82) | 88(4.40) | 88(4.48) | 60(3.26) |  |
| Unmarried | 16(0.75) | 17(0.85) | 12(0.61) | 14(0.76) |  |
| **Education** |  |  |  |  | <0.001 |
| Junior high school or below | 156(7.36) | 160(7.99) | 167(8.50) | 179(9.71) |  |
| High school | 353(16.65) | 329(16.43) | 356(18.13) | 381(20.67) |  |
| College or above | 1611(75.99) | 1513(75.57) | 1441(73.37) | 1283(69.61) |  |
| **Occupation** |  |  |  |  | <0.001 |
| Mental work | 1322(62.36) | 1191(59.49) | 1131(57.59) | 991(53.77) |  |
| Physical labor | 549(25.90) | 528(26.37) | 535(27.24) | 554(30.06) |  |
| Unemployed | 249(11.75) | 283(14.14) | 298(15.17) | 298(16.17) |  |
| **Family history of diabetes** |  |  |  |  | 0.001 |
| Yes | 291(13.73) | 296(14.79) | 343(17.46) | 323(17.53) |  |
| No | 1829(86.27) | 1706(85.21) | 1621(82.54) | 1520(82.47) |  |
| **Age of menarche** |  |  |  |  | 0.007 |
| <12 years old | 320(15.09) | 284(14.19) | 313(15.94) | 280(15.19) |  |
| ≥12 years old | 1743(82.22) | 1664(83.12) | 1574(80.14) | 1480(80.30) |  |
| Not sure | 57(2.69) | 54(2.70) | 77(3.92) | 83(4.50) |  |
| **Menopause status** |  |  |  |  | <0.001 |
| Premenopause | 959(45.24) | 841(42.01) | 756(38.49) | 610(33.10) |  |
| Postmenopause | 1161(54.76) | 1161(57.99) | 1208(61.51) | 1233(66.90) |  |
| **Age at first childbirth** |  |  |  |  | 0.940 |
| ≤20 or＞35 years old | 87(4.10) | 75(3.75) | 75(3.82) | 71(3.85) |  |
| 21-35 years old | 2033(95.90) | 1927(96.25) | 1889(96.18) | 1772(96.15) |  |
| **Breastfeeding time** |  |  |  |  | <0.001 |
| <6 months | 490(23.11) | 401(20.03) | 342(17.41) | 336(18.23) |  |
| ≥6 months | 1325(62.50) | 1300(64.94) | 1328(67.62) | 1216(65.98) |  |
| No breastfeeding | 305(14.39) | 301(15.03) | 294(14.97) | 291(15.79) |  |
| **Gestational diabetes** |  |  |  |  | 0.070 |
| Yes | 32(1.51) | 39(1.95) | 36(1.83) | 49(2.66) |  |
| No | 2088(98.49) | 1963(98.05) | 1928(98.17) | 1794(97.34) |  |
| **Gestational hypertension** |  |  |  |  | 0.124 |
| Yes | 75(3.54) | 62(3.10) | 54(2.75) | 75(4.07) |  |
| No | 2045(96.46) | 1940(96.90) | 1910(97.25) | 1768(95.93) |  |
| **Smoking status** |  |  |  |  | . |
| Never-smoker | 2033(95.90) | 1933(96.55) | 1884(95.93) | 1760(95.50) |  |
| Current-smoker | 15(0.71) | 13(0.65) | 25(1.27) | 31(1.68) |  |
| Ex-smoker | 3(0.14) | 4(0.20) | 4(0.20) | 6(0.33) |  |
| Involuntary-smoker | 69(3.25) | 52(2.60) | 51(2.60) | 46(2.50) |  |
| **Drinking status** |  |  |  |  | 0.043 |
| Never-drinker | 1936(91.32) | 1847(92.26) | 1830(93.18) | 1718(93.22) |  |
| Current-drinker | 180(8.49) | 145(7.24) | 129(6.57) | 116(6.29) |  |
| Ex-drinker | 4(0.19) | 10(0.50) | 5(0.25) | 9(0.49) |  |
| **Exercise or not** |  |  |  |  | 0.001 |
| Yes | 1682(79.34) | 1560(77.92) | 1484(75.56) | 1372(74.44) |  |
| No | 438(20.66) | 442(22.08) | 480(24.44) | 471(25.56) |  |
| BMI (Kg/m2) | 21.74(20.41,23.16) | 22.50(21.18,24.20) | 23.20(21.63,24.80) | 23.88(22.20,25.70) | <0.001 |
| **WC (cm)** | 73.00(69.00,77.00) | 76.00(72.00,80.00) | 78.00(73.00,82.00) | 80.00(75.00,84.00) | <0.001 |
| **HC (cm)** | 91.00(88.00,94.00) | 92.00(89.00,95.00) | 92.00(89.00,96.00) | 93.00(90.00,96.50) | <0.001 |
| **SBP (mmHg)** | 114.00(106.00,126.00) | 117.00(107.00,128.00) | 120.00(109.00,130.00) | 122.00(112.00,133.00) | <0.001 |
| **DBP (mmHg)** | 69.00(62.00,77.00) | 70.00(64.00,78.00) | 72.00(66.00,80.00) | 74.00(66.00,82.00) | <0.001 |
| **FPG (mmol/L)** | 5.10(4.80,5.40) | 5.18(4.90,5.46) | 5.24(4.94,5.55) | 5.32(5.02,5.65) | <0.001 |
| **TC (mmol/L)** | 5.16(4.62,5.72) | 5.30(4.72,5.89) | 5.38(4.77,6.03) | 5.52(4.88,6.20) | <0.001 |
| **TG (mmol/L)** | 0.76(0.64,0.87) | 1.07(0.95,1.20) | 1.42(1.27,1.59) | 2.22(1.88,2.77) | <0.001 |
| **HDL-C (mmol/L)** | 1.80(1.61,2.00) | 1.60(1.45,1.75) | 1.44(1.31,1.59) | 1.26(1.14,1.40) | <0.001 |
| **LDL-C (mmol/L)** | 2.94(2.48,3.43) | 3.18(2.67,3.71) | 3.24(2.72,3.81) | 3.13(2.53,3.72) | <0.001 |
| **ALT (U/L)** | 17.00(13.00,22.00) | 18.00(14.00,23.00) | 19.00(15.00,25.00) | 21.00(16.00,29.00) | <0.001 |
| **BUN (mmol/L)** | 4.56(3.87,5.39) | 4.44(3.82,5.28) | 4.45(3.78,5.24) | 4.41(3.72,5.19) | <0.001 |
| **Scr (μmol/L)** | 58.00(52.00,64.00) | 58.00(53.00,65.00) | 58.00(53.00,64.00) | 59.00(53.00,65.00) | 0.031 |
| **UA (μmol/L)** | 255.00(225.00,288.00) | 267.00(236.00,304.00) | 284.50(250.00,319.25) | 306.00(269.00,343.00) | <0.001 |
| **Prediabetes** |  |  |  |  | <0.001 |
| Yes | 280(13.21) | 339(16.93) | 434(22.10) | 539(29.25) |  |
| No | 1840(86.79) | 1663(83.07) | 1530(77.90) | 1304(70.75) |  |

AIP: atherogenic index of plasma, ALT: alanine aminotransferase, BMI: body mass index, BUN: blood urea nitrogen, DBP: diastolic blood pressure, FPG: fasting plasma glucose, HC: hip circumference, HDL-C: high-density lipoprotein cholesterol, LDL-C: low-density lipoprotein cholesterol, SBP: systolic blood pressure, Scr: serum creatinine, TC: total cholesterol, TG: triglycerides, UA: uric acid, WC: waist circumference
