# Supplementary material for: Does menopause influence the association between atherogenic index of plasma and prediabetes? A cross-sectional study in middle-aged Chinese women
Source: PLoS One. 2026 Feb 12;21(2):e0342644. doi: 10.1371/journal.pone.0342644 (PMC12900311; doi:10.1371/journal.pone.0342644)
Supplement: S4 Appendix — (DOCX) [file pone.0342644.s004.docx]

**S4 Appendix**

**Table S3** Comparison of baseline characteristics of the included and excluded participants.

| **Variables** | **ALL**  **(n=12,885)** | **Excluded**  **(n=4,956)** | **Included**  **(n=7,929)** | ***P*** |
| --- | --- | --- | --- | --- |
| **Glucose metabolism status** |  |  |  | <0.001 |
| Prediabetes | 3046(23.64) | 1454(29.34) | 1592(20.08) |  |
| Non-prediabetes | 9839(76.36) | 3502(70.66) | 6337(79.92) |  |
| **Age (years)** | 53.00(49.00,59.00) | 61.00(52.00,64.00) | 52.00(48.00,54.00) | <0.001 |
| **Marital status** |  |  |  | <0.001 |
| Married | 11861(94.13) | 4308(92.23) | 7553(95.26) |  |
| Divorced or widowed | 594(4.71) | 277(5.93) | 317(4.00) |  |
| Unmarried | 145(1.15) | 86(1.84) | 59(0.74) |  |
| **Education** |  |  |  | <0.001 |
| Junior high school or below | 1187(9.88) | 525(12.85) | 662(8.35) |  |
| High school | 2748(22.87) | 1329(32.53) | 1419(17.90) |  |
| College or above | 8080(67.25) | 2232(54.63) | 5848(73.75) |  |
| **Occupation** |  |  |  | <0.001 |
| Mental work | 6258(49.57) | 1623(34.57) | 4635(58.46) |  |
| Physical labor | 3689(29.22) | 1523(32.44) | 2166(27.32) |  |
| Unemployed | 2677(21.21) | 1549(32.99) | 1128(14.23) |  |
| **Family history of diabetes** |  |  |  | <0.001 |
| Yes | 1850(14.66) | 597(12.73) | 1253(15.80) |  |
| No | 10767(85.34) | 4091(87.27) | 6676(84.20) |  |
| **Age of menarche** |  |  |  | <0.001 |
| <12 years old | 1901(15.06) | 704(15.00) | 1197(15.10) |  |
| ≥12 years old | 10189(80.72) | 3728(79.44) | 6461(81.49) |  |
| Not sure | 532(4.21) | 261(5.56) | 271(3.42) |  |
| **Menopausal status** |  |  |  | <0.001 |
| Premenopause | 4053(32.18) | 887(19.01) | 3166(39.93) |  |
| Postmenopause | 8542(67.82) | 3779(80.99) | 4763(60.07) |  |
| **Age at first childbirth** |  |  |  | <0.001 |
| ≤20 or＞35 years old | 470(3.75) | 162(3.52) | 308(3.88) |  |
| 21-35 years old | 11812(94.24) | 4191(91.01) | 7621(96.12) |  |
| No offspring | 252(2.01) | 252(5.47) | 0(0.00) |  |
| **Breastfeeding time** |  |  |  | 0.093 |
| <6 months | 2471(20.12) | 902(20.72) | 1569(19.79) |  |
| ≥6 months | 7922(64.50) | 2753(63.24) | 5169(65.19) |  |
| No breastfeeding | 1889(15.38) | 698(16.03) | 1191(15.02) |  |
| **Gestational diabetes** |  |  |  | 0.374 |
| Yes | 252(2.05） | 96(2.21) | 156(1.97) |  |
| No | 12030(97.95) | 4257(97.79) | 7773(98.03) |  |
| **Gestational hypertension** |  |  |  | <0.001 |
| Yes | 488(3.97) | 222(5.10) | 266(3.35) |  |
| No | 11794(96.03) | 4131(94.90) | 7663(96.65) |  |
| **Smoking status** |  |  |  | 0.003 |
| Never-smoker | 12140(96.17) | 4530(96.49) | 7610(95.98) |  |
| Current-smoker | 153(1.21) | 69(1.47) | 84(1.06) |  |
| Ex-smoker | 25(0.20) | 8(0.17) | 17(0.21) |  |
| Involuntary-smoker | 306(2.42) | 88(1.87) | 218(2.75) |  |
| **Drinking status** |  |  |  | 0.037 |
| Never-drinker | 11728(92.90) | 4397(93.65) | 7331(92.46) |  |
| Current-drinker | 852(6.75) | 282(6.01) | 570(7.19) |  |
| Ex-drinker | 44(0.35) | 16(0.34) | 28(0.35) |  |
| **Exercise or not** |  |  |  | <0.001 |
| Yes | 9850(78.03) | 3752(79.91) | 6098(76.91) |  |
| No | 2774(21.97) | 943(20.09) | 1831(23.09) |  |
| **BMI (Kg/m2)** | 22.90(21.30,24.71) | 23.17(21.49,25.13) | 22.76(21.20,24.50) | <0.001 |
| **WC (cm)** | 77.00(72.00,82.00) | 78.00(73.00,84.00) | 76.00(72.00,81.00) | <0.001 |
| **HC (cm)** | 92.00(89.00,95.30) | 92.00(89.00,96.00) | 92.00(89.00,95.00) | 0.029 |
| **SBP (mmHg)** | 120.00(110.00,132.00) | 126.00(114.00,136.00) | 118.00(108.00,130.00) | <0.001 |
| **DBP (mmHg)** | 72.00(66.00,80.00) | 73.00(66.00,80.00) | 72.00(64.00,80.00) | <0.001 |
| **FPG (mmol/L)** | 5.26(4.95,5.59) | 5.36(5.04,5.72) | 5.21(4.91,5.51) | <0.001 |
| **TC (mmol/L)** | 5.36(4.76,6.00) | 5.42(4.81,6.08) | 5.33(4.73,5.96) | <0.001 |
| **TG (mmol/L)** | 1.24(0.92,1.73) | 1.30(0.96,1.81) | 1.21(0.89,1.68) | <0.001 |
| **HDL-C (mmol/L)** | 1.52(1.33,1.74) | 1.52(1.32,1.75) | 1.52(1.33,1.74) | 0.849 |
| **LDL-C (mmol/L)** | 3.13(2.59,3.69) | 3.15(2.58,3.72) | 3.11(2.60,3.66) | 0.167 |
| **ALT (U/L)** | 19.00(14.00,25.00) | 19.00(15.00,25.00) | 18.00(14.00,24.00) | <0.001 |
| **BUN (mmol/L)** | 4.56(3.87,5.37) | 4.69(4.01,5.50) | 4.48(3.80,5.28) | <0.001 |
| **Scr (μmol/L)** | 58.00(53.00,65.00) | 58.00(53.00,65.00) | 58.00(53.00,64.00) | 0.290 |
| **UA (μmol/L)** | 279.00(242.00,320.00) | 284.00(243.00,329.00) | 276.00(241.00,314.00) | <0.001 |
| **AIP** | -0.21(-0.59,0.21) | -0.16(-0.55,0.26) | -0.24(-0.63,0.17) | <0.001 |
| **AIP quartile** |  |  |  | <0.001 |
| Q1 | 3217(25.01) | 1097(22.22) | 2120(26.74) |  |
| Q2 | 3216(25.00) | 1214(24.59) | 2002(25.25) |  |
| Q3 | 3216(25.00) | 1252(25.36) | 1964(24.77) |  |
| Q4 | 3216(25.00) | 1373(27.82) | 1843(23.24) |  |

AIP: atherogenic index of plasma, ALT: alanine aminotransferase, BMI: body mass index, BUN: blood urea nitrogen, DBP: diastolic blood pressure, FPG: fasting plasma glucose, HC: hip circumference, HDL-C: high-density lipoprotein cholesterol, LDL-C: low-density lipoprotein cholesterol, SBP: systolic blood pressure, Scr: serum creatinine, TC: total cholesterol, TG: triglycerides, UA: uric acid, WC: waist circumference
