# Supplementary material for: Does menopause influence the association between atherogenic index of plasma and prediabetes? A cross-sectional study in middle-aged Chinese women
Source: PLoS One. 2026 Feb 12;21(2):e0342644. doi: 10.1371/journal.pone.0342644 (PMC12900311; doi:10.1371/journal.pone.0342644)
Supplement: S5 Appendix — (DOCX) [file pone.0342644.s005.docx]

**S5 Appendix**

**Table S4** Sensitivity analysis of the association between AIP and prediabetes.

| **Variables** | **Participants**  **(Prediabetes)** | **Crude model** | | **Model 1** | | **Model 2** | |
| --- | --- | --- | --- | --- | --- | --- | --- |
|  |  | **OR (95%CI)** | ***P*** | **OR (95%CI)** | ***P*** | **OR (95%CI)** | ***P*** |
| AIP | 9,503(1,908) | 1.75(1.62,1.90) | <0.001 | 1.69(1.55,1.83) | <0.001 | 1.40(1.27,1.55) | <0.001 |
| AIP | 7,929(185) | 1.87(1.51,2.31) | <0.001 | 1.75 (1.40,2.19) | <0.001 | 1.71(1.27,2.32) | <0.001 |
| AIP | 7,929(1,579) | 1.81(1.65,1.98) | <0.001 | 1.74(1.59,1.91) | <0.001 | 1.46(1.31,1.63) | <0.001 |
| AIP | 7,892(1,586) | 1.80(1.64,1.96) | <0.001 | 1.73(1.58,1.89) | <0.001 | 1.45(1.30,1.61) | <0.001 |

AIP: atherogenic index of plasma, CI: confidence interval, OR: odds ratio.

The four rows of AIP data represent the results from four distinct sensitivity analyses:

Analysis 1 (Top row): Included all participants without exclusion for missing data, with multiple imputation applied to handle missing variables.

Analysis 2 (Second row): Redefined prediabetes using a FPG diagnostic threshold of 6.1 to 6.9 mmol/L.

Analysis 3 (Third row): Excluded individuals who self-reported a prior diagnosis of prediabetes.

Analysis 4 (Bottom row): Further excluded participants who were using hormonal medications.

The Crude model was not adjusted for any covariates.

Model 1 was adjusted for age, education, marital status, occupation, smoking status, drinking status, exercise status, family history of diabetes, age of menarche, age at first childbirth, breastfeeding time, history of gestational diabetes, and gestational hypertension.

Model 2 was also adjusted for BMI, SBP, DBP, WC, HC, TC, LDL-C, ALT, BUN, Scr, and UA based on Model 1.
