# Supplementary material for: Does menopause influence the association between atherogenic index of plasma and prediabetes? A cross-sectional study in middle-aged Chinese women
Source: PLoS One. 2026 Feb 12;21(2):e0342644. doi: 10.1371/journal.pone.0342644 (PMC12900311; doi:10.1371/journal.pone.0342644)
Supplement: S6 Appendix — (DOCX) [file pone.0342644.s006.docx]

**S6 Appendix**

Table S5 Sensitivity analysis for the joint association of AIP and menopause on prediabetes.

| **Variables** | **Participants** | **Crude model** | | **Model 1** | | **Model 2** | |
| --- | --- | --- | --- | --- | --- | --- | --- |
|  |  | **OR (95% CI)** | ***P*** | **OR (95% CI)** | ***P*** | **OR (95% CI)** | ***P*** |
| Low AIP-premenopause | 1908 | Reference |  | Reference |  | Reference |  |
| Low AIP -postmenopause | 2508 | 1.43 (1.21,1.70) | <0.001 | 1.18(0.97,1.43) | 0.097 | 1.12 (0.92,1.37) | 0.244 |
| High AIP-premenopause | 1258 | 2.06 (1.71,2.49) | <0.001 | 2.00 (1.65,2.42) | <0.001 | 1.54 (1.26,1.88) | <0.001 |
| High AIP-postmenopause | 2255 | 2.66 (2.26,3.13) | <0.001 | 2.13 (1.75,2.58) | <0.001 | 1.64 (1.34,2.01) | <0.001 |

AIP was divided into the Low AIP group and the High AIP group with -0.16 as the cutoff value.

AIP: atherogenic index of plasma, CI: confidence interval, OR: odds ratio.

The Crude model was not adjusted for any covariates.

Model 1 was adjusted for age, education, marital status, occupation, smoking status, drinking status, exercise status, family history of diabetes, age of menarche, age at first childbirth, breastfeeding time, history of gestational diabetes, and gestational hypertension.

Model 2 was also adjusted for BMI, SBP, DBP, WC, HC, TC, LDL-C, ALT, BUN, Scr, and UA based on Model 1.
