# Supplementary material for: Does menopause influence the association between atherogenic index of plasma and prediabetes? A cross-sectional study in middle-aged Chinese women
Source: PLoS One. 2026 Feb 12;21(2):e0342644. doi: 10.1371/journal.pone.0342644 (PMC12900311; doi:10.1371/journal.pone.0342644)
Supplement: Attachment — Submitted filename: Response to Reviewers.docx [file pone.0342644.s008.docx]

Dear Editor and Reviewers:

Thank you for your correspondence and the insightful comments regarding our manuscript “Does menopause influence the association between atherogenic index of plasma and prediabetes? A cross-sectional study in middle-aged Chinese women” (ID: PONE-D-25-48687). Based on your constructive suggestions, we have revised our manuscript, and our detailed responses to each comment are provided below. To facilitate your review, we have quoted the revised text from the manuscript directly in this response letter and presented it in red font.

**Reviewer #1:**

**Comment 1:**Multivariate analysis needs to be done to find the association between AIP and prediabetes.

**Response:**We thank the reviewer for the comment. As suggested, multivariate logistic regression analysis was performed to find the association between AIP and prediabetes. This analysis was conducted not only for the entire study population but also specifically stratified by menopausal status (pre-menopausal and post-menopausal middle-aged women) to explore potential effect modifications. The requested multivariate analysis has been conducted and was shown in Table 2.

“We established three models for the regression analyses: The crude model did not adjust for any covariate, Model 1 was adjusted for sociodemographic information, and Model 2 was additionally adjusted for physical examinations and laboratory tests, excluding TG and HDL-C.”(Method:Statistical analysis)

“In all three models, the continuous AIP and prediabetes showed a significant positive association, with an OR of 1.45 in the final model (95% CI: 1.30,1.62). In the AIP quartile groups, the risk of prediabetes increased with each increasing AIP quartile in all three models (*P* for trend<0.001, for all). In the fully adjusted Model 2, compared to the lowest quartile (Q1), the adjusted Ors (95% CI) for prediabetes in the Q2, Q3, and Q4 groups were 1.20 (1.01, 1.43), 1.48 (1.24, 1.77), and 1.83 (1.53,2.20), respectively. Similarly, in the subgroup analysis stratified by menopausal status, AIP (continuous/quartile groups) and prediabetes showed significant positive association in both premenopausal women and postmenopausal women.”(Results: The association between AIP and prediabetes)

**Comment** 2:Few grammatical errors need to be rectified.

**Response:**We appreciate the reviewer's feedback. The manuscript has been carefully reviewed by a professional language editing service to ensure grammatical accuracy.

**Comment 3:**P value table 1 can be simplified.

**Response:** We thank the reviewer for the suggestion. The P-values in Table 1 have been simplified and are now consistently presented to three decimal places, with *P* < 0.001 being used for significant results below this threshold.

**Comment 4:**The reference for the diagnosis of the prediabetes can be given

**Response:**We thank the reviewer for the suggestion. The reference for the American Diabetes Association diagnostic criteria for prediabetes has been added as reference number 29.

“The outcome variable, prediabetes, was defined based on the American Diabetes Association criteria as either 5.6 mmol/L≤ FPG<7.0 mmol/L or self-reported prediabetes (29).”(Methods: Data collection)

**Reviewer #2:**

**Comment 1:**I believe you should make data and code publicly available and revise the Data Availability Statement to meet PLOS policy.

**Response:**We have revised the Data Availability Statement as suggested. The data are not yet publicly available as they are part of ongoing studies. However, in full compliance with PLOS policy, the data underlying this study are available from the corresponding authors upon reasonable request.

**Comment 2:**Please add and report the AIP×menopausal status interaction (estimate, 95% CI, p-value), with marginal effects plots.

**Response:**We thank the reviewer for this insightful suggestion. Following the reviewer's advice, we have formally tested the multiplicative interaction between AIP and menopausal status in the fully adjusted model.

“To quantitatively assess the moderating role of menopausal status, we introduced an interaction term (AIP × menopausal status) into the fully adjusted logistic regression model (Model 2). The analysis revealed no statistically significant multiplicative interaction between AIP and menopausal status on the risk of prediabetes (OR for interaction = 1.00, 95% CI: 0.83, 1.22; P =0.973).” (Results:Interaction between AIP and menopausal status)

Given the non-significant interaction result, a marginal effects plot would primarily illustrate the absence of an effect, which is already clearly communicated by the reported statistical values. We believe that the stratified analyses (Table 2) and the joint effect analysis (Table 3) more meaningfully capture the relationship patterns in the data. Therefore, we have not included the plot to avoid redundancy and to maintain the conciseness of the manuscript.

**Comment 3:**Conduct sensitivity analyses for outcome definition.Exclude self-report only; alternative FPG thresholds; subset with HbA1c if available

**Response:**Thank you for the suggestion. We have conducted sensitivity analyses to address this: (1) Excluded self-reported diabetes only; (2) Used alternative FPG thresholds (6.0 and 7.8 mmol/L).However, regarding HbA1c, it was not routinely collected, leading to substantial missing data. Therefore, a formal sensitivity analysis for pre-diabetes was not feasible. All findings confirmed the robustness of our primary results.

“To assess the robustness of the association between the atherogenic index of plasma (AIP) and prediabetes, we conducted four sensitivity analyses, as summarized in Table S4 (S5 Appendix) . The first analysis retained all participants without excluding those with missing data and applied multiple imputation for missing variables. The second analysis redefined prediabetes using a diagnostic threshold of 6.1 ≤ FPG < 7.0 mmol/L. The third analysis excluded individuals who self-reported a diagnosis of prediabetes. The fourth analysis further excluded participants using hormonal medications. Across all four analyses, AIP remained significantly and positively associated with prediabetes (all *P* < 0.001). Although the effect sizes varied somewhat across models, the overall trend was consistent, indicating that the association between AIP and prediabetes is robust under different analytical scenarios.” (Results:Sesitivity analyis and S5 Appendix)

**Comment 4:**Address medication and MHT confounding (adjust or explicitly discuss and run sensitivity analyses).

**Response:** Thank you for raising this important point. We acknowledge the potential for confounding by medication use, including MHT. As the number of users was small, these individuals were initially retained in the primary analysis to preserve statistical power. Therefore, we performed a sensitivity analysis by excluding all participants using relevant medications (e.g., MHT, hormonal drugs). The results from this analysis were consistent with our primary findings, confirming that the observed associations were robust and not substantially confounded by medication use.

“The fourth analysis further excluded participants using hormonal medications. Across all four analyses, AIP remained significantly and positively associated with prediabetes (all *P* < 0.001).” (Results:Sesitivity analyis and S5 Appendix)

**Comment 5:**You should report missing data proportions, outlier criteria, comparisons of included vs. excluded, and consider multiple imputation where appropriate.

**Response:**We thank the reviewer for these valuable methodological suggestions. We have now comprehensively addressed these points in the revised manuscript.

(1) The proportions of missing data for key variables are reported in S2 Appendix. As pre-specified, variables with >10% missingness were excluded from the primary analysis. To address potential selection bias arising from these missingness, we performed multiple imputation as a sensitivity analysis . The results were consistent with our primary complete-case analysis, confirming the robustness of our findings against these expected differences.

“The primary analysis employed complete-case approach after assessing missing data patterns, variables with >10% missing values were excluded. Table S1 (S2 Appendix) showed the missing data proportions.” (Methods:Statistical analysis and S2 Appenix)

“The first analysis retained all participants without excluding those with missing data and applied multiple imputation for missing variables......Across all four analyses, AIP remained significantly and positively associated with prediabetes (all *P* < 0.001).” (Results:Sesitivity analyis and S5 Appendix)

(2) The criterion for outlier handling (Tukey's method, 1.5×IQR) is now explicitly stated in the 'Statistical analysis' section; all identified outliers were retained as they represented biologically plausible values.

“Outliers were identified using Tukey's method (1.5×IQR) and retained unless erroneous.” (Methods: Statistical analysis)

(3) A comparison between included and excluded participants has been conducted and is detailed in S4 Appendix. As expected, given that our study specifically focuses on a middle-aged women and age was the primary exclusion criterion, there were statistically significant differences in several baseline characteristics. This is a direct consequence of our cohort definition.

**Comment 6:**Necessary to provide model diagnostics (linearity, collinearity, fit/influence) and effect-size scaling for AIP.

**Response:**We thank the reviewer for this important suggestion. We have now performed comprehensive model diagnostics as requested.

(1) We assessed multicollinearity by calculating the VIFs for all covariates in the logistic regression model. All VIF values were well below the threshold of 10, indicating that multicollinearity was not a major concern in our models.

“VIFs for all variables in the fully adjusted model were below 10.”(Results:The association between AIP and prediabetes)

(2) Regarding effect-size scaling, the association was already assessed using AIP quartiles, which provides a clinically interpretable effect size by comparing risk across population percentiles. The consistent, graded increase in odds ratios across quartiles confirms a robust and interpretable association.

“In the AIP quartile groups, the risk of prediabetes increased with each increasing AIP quartile in all three models (P for trend<0.001, for all). In the fully adjusted Model 2, compared to the lowest quartile (Q1), the adjusted Ors (95% CI) for prediabetes in the Q2, Q3, and Q4 groups were 1.20 (1.01, 1.43), 1.48 (1.24, 1.77), and 1.83 (1.53,2.20), respectively.” (Results:The association between AIP and prediabetes)

**Comment 7:**RCS knot reference value and may need justify any exposure dichotomization or present continuous joint effects.

**Response:**We thank the reviewer for this valuable input. Our decision to dichotomize AIP served two purposes: (1) to enable the identification of clinically actionable subgroups (e.g., the 'High-AIP Postmenopausal' group), and (2) to utilize a statistically-derived threshold for risk categorization. Specifically, the cutoff of 0.24 was derived from the RCS model as the point where the AIP-prediabetes association crossed the null value (OR=1), ensuring the resulting categories reflect meaningful differences in risk.

**Comment 8:**Clarify fasting status, analytic platforms, QC; correct typographical and figure export issues.

**Response:**We thank the reviewer for these important points regarding methodological rigor. We have revised the manuscript to address each concern as follows:

(1) Fasting Status & QC Procedures: The Methods section now explicitly states that all blood samples were collected after an overnight fast of at least 8 hours. Furthermore, we have detailed our rigorous quality control (QC) protocol, which included standardized training for all data collectors and duplicate data entry with cross-verification to ensure maximal accuracy.

“All blood samples were collected after an overnight fast of at least 8 hours.” (Methods: Data collection)

(2) Analytic Platforms: All laboratory analyses were performed using the standardized clinical laboratory platforms at our tertiary hospital, which remained consistent throughout the study period. To maintain conciseness and focus, and given the single-center, standardized nature of our laboratory methods, specific instrument models have been omitted, as the assays are considered routine and well-standardized.

(3) Formatting Issues:The manuscript has been thoroughly proofread to correct typographical errors. All figures have been re-exported at high resolution to ensure clarity.

**Comment 9:**Improve consistency between Methods and Discussion.

**Response:** We thank the reviewer for highlighting the importance of consistency. In response, we have meticulously revised the Discussion section to ensure that the interpretation of our findings strictly aligns with the statistical approaches predefined in the Methods. Specifically, the sequence and framing of our arguments in the Discussion now directly reflect the order and intent of the analyses conducted, such as the use of multivariate logistic regression for association, stratification by menopausal status, and sensitivity analyses for robustness. This ensures a logical and consistent narrative from our planned methods through to the interpretation of results.

**Comment 10:**Correcttypos (e.g.,“methodaology”-“methodology”) and address garbled figure labels.

**Response:** We apologize for these errors. The manuscript has been meticulously proofread to correct all typographical errors, including the noted "methodaology". Figure labels also have been reviewed to ensure clarity and correct display.

**Reviewer #3:**

**Comment 1:**The authors propose using the Atherogenic Index of Plasma (AIP) as a novel biomarker for prediabetes in a retrospective analysis of 7,929 middle-aged women in China.The exclusions for the analysis included a diagnosis of diabetes (n=248) in line #108.This corresponds to 1.9% of the initial sample, which is lower than the reported 13.7% by Zhou et al. (2023).The authors need to explain this discrepancy and whether this might be a limitation or an advantage. In addition, why use the AIP if we already have a direct marker (A1C)?

**Response:** We thank the reviewer for these insightful observations regarding our study population and the rationale for investigating AIP.

(1) We acknowledge that the baseline prevalence of diabetes in our cohort (1.9%) is lower than the national average (13.7%) reported by Zhou et al. This discrepancy is likely attributable to the nature of our study population, which was recruited from a health examination center and may thus represent a more health-conscious group, such as employees undergoing routine occupational health checks. Although this characteristic may limit the generalizability of our prevalence estimates, it also strengthens the internal validity of our study for investigating dysglycemia progression, as the lower baseline disease burden allows a clearer assessment of the transition to prediabetes. In line with the reviewer’s comment, we have explicitly acknowledged this aspect as a limitation in the revised manuscript.

“Second, this single-center study of middle-aged Chinese women recruited from a tertiary hospital in Changsha City may limit the generalizability of our findings to other settings, regions, ethnicities, and age groups.”(Discussion: Paragraph 6)

(2) We investigated AIP not to replace HbA1c, but as a complementary, mechanistic marker. AIP reflects atherogenic dyslipidemia and insulin resistance—the core pathophysiology preceding hyperglycemia—offering earlier risk stratification from a routine lipid panel.

“Unlike glycosylated hemoglobin which indicates established dysglycemia, AIP provides complementary pathophysiological information on the underlying metabolic disturbances that often precede hyperglycemia, making it valuable for early risk stratification. ”(Introduction:Paragraph 2)

**Reviewer #4:** 
**Comment 1:**The retrospective cross-sectional design precludes establishing temporality and causal inference. The authors acknowledge this limitation but should more emphatically describe this constraint in the abstract, introduction, methods, and discussion. This is critical to appropriately frame the findings as associations, not causations.
**Response:** We thank the reviewer for this critical point. We have emphatically reframed the language throughout the manuscript to consistently describe our findings as associations and to preclude any causal interpretation. The limitations of the cross-sectional design are now more strongly highlighted.

“First, the retrospective cross-sectional design fundamentally precludes the establishment of temporality and causal inference between AIP and prediabetes, so these findings should be interpreted strictly as correlational relationships rather than evidence of causation.”(Discussion: Paragraph 6)

**Comment 2:**Mediation analysis presented (if any) is inappropriate given cross-sectional data, as temporal ordering of variables is unknown. The authors should clarify or consider reframing such analysis as moderation or effect modification.
**Response:**We thank the reviewer for their careful reading. We wish to clarify that our study did not conduct any mediation analysis. The analyses presented are focused on assessing the association and potential effect modification (interaction) by menopausal status, not mediation. We apologize for any confusion our wording may have caused.

**Comment 3:**The authors excluded participants with missing data rather than using multiple imputation, which may lead to biased results if data are not Missing Completely at Random, and I recommend providing justification for this approach or conducting sensitivity analyses using principled missing data methods to improve validity.
**Response:**We thank the reviewer for this important methodological point. In response, we have conducted a sensitivity analysis using multiple imputation for missing data. The results were consistent with our primary complete-case analysis, confirming the robustness of our findings.

“The first analysis retained all participants without excluding those with missing data and applied multiple imputation for missing variables...... Across all four analyses, AIP remained significantly and positively associated with prediabetes (all *P* < 0.001).”(Results:Sensitivity analysis and S5 Appendix).

**Comment 4:**The manuscript does not provide a formal sample size calculation or power analysis, limiting the ability to assess whether the study was adequately powered to detect significant associations, particularly in subgroup and interaction analyses. (Include sample size justification or power analysis if possible.)
**Response:**We thank the reviewer for raising this important point. As a retrospective analysis of an existing cohort, our sample size was determined by the available data rather than a pre-specified power calculation. However, our final analytical cohort of 7,929 participants is substantially larger than those in many similar epidemiological studies.

**Comment 5:**Provide more detailed explanation of inclusion/exclusion criteria, including how comorbidities were identified.
**Response:**We sincerely thank the reviewer for this insightful suggestion. To present the participant selection process more clearly and concisely, we included a participant flow diagram (Figure 1) in the manuscript, which visually outlines the inclusion and exclusion steps.

**Comment 6:**Though many confounders are controlled for, significant potential confounders such as insulin resistance, sex hormone levels, and duration/timing of menopause are not measured. These could substantially bias results and should be discussed in more detail.
**Response:**We thank the reviewer for this crucial point. We fully agree that unmeasured confounders such as insulin resistance and sex hormone levels could bias the results. As suggested, we have now explicitly discussed this limitation in greater detail within the 'Limitations' section of the Discussion.

“Third, we lacked data on important potential confounders including insulin resistance, sex hormone levels, and detailed menopausal characteristics, which may introduce residual confounding.”(Discussion: Paragraph 6)

**Comment 7:**The reliance on self-reported menopausal status and retrospective electronic records introduces misclassification and information biases. The discussion section would benefit from explicitly addressing these biases and their possible impact on the findings.
**Response:**We thank the reviewer for raising this important point. We have now explicitly acknowledged the potential for misclassification and information biases due to self-reported menopausal status and retrospective records in the Limitations section, and have discussed their possible impact on the findings.

“Fourth, some data relied on self-report and is subject to information bias.”(Discussion: Paragraph 6)

**Comment 8:**Selection bias is possible given hospital-based convenience sampling. Generalizability to broader populations is limited and should be acknowledged with more emphasis.

**Response:**We thank the reviewer for this comment. We have now emphasized the potential for selection bias and the limited generalizability more explicitly in the revised Limitations section.

“Second, this single-center study of middle-aged Chinese women recruited from a tertiary hospital in Changsha City may limit the generalizability of our findings to other settings, regions, ethnicities, and age groups.”(Discussion: Paragraph 6)

**Comment 9:**While the biological plausibility linking menopausal estrogen decline to dysregulated lipid and glucose metabolism is well-discussed, the mechanistic elaboration could be deeper, citing recent advances on hormonal regulation.
**Response:**We thank the reviewer for this insightful suggestion. We have deepened the mechanistic elaboration in the discussion by including recent advances on how estrogen deficiency affects adipose tissue function, systemic inflammation, and direct regulatory roles in pancreatic and hepatic glucose metabolism, supported by additional references.

“However, we observed a numerical pattern where the association was most pronounced in postmenopausal women with high AIP levels. This pattern is biologically plausible, as menopause is accompanied with the decline in ovarian function and changes in hormonal levels, which can affect lipid and glucose metabolism (35, 36). Beyond the well-known effects on insulin secretion and sensitivity(37, 38), recent advances highlight that estrogen deficiency impairs adipose tissue function, promoting a pro-inflammatory state and the release of free fatty acids, which further exacerbates insulin resistance and dysglycemia (39). Additionally, estrogen receptors in the pancreas and liver play direct roles in beta-cell survival and hepatic glucose output, the dysfunction of which post-menopause contributes significantly to metabolic dysregulation (40).”(Discussion: Paragraph 4)

**Comment 10:**The authors’ claims about AIP’s clinical utility as a screening marker should be tuned down, stressing the need for prospective validation in independent cohorts before clinical translation.
**Response:**We thank the reviewer for this suggestion. We have revised the discussion to tone down our claims regarding the clinical utility of AIP, now emphasizing that its use as a screening marker requires prospective validation in independent cohorts.

“Our findings highlight the potential of AIP as a cost-effective biomarker for identifying individuals at risk; however, its clinical utility as a screening tool requires further validation in prospective studies.”(Discussion: Paragraph 2)

**Comment 11:**Some parts of the discussion are somewhat repetitive and could benefit from more concise phrasing.
**Response:**We thank the reviewer for this valuable feedback. We have thoroughly revised the Discussion section to eliminate repetitive statements and improve conciseness.

**Comment 12:**The discussion could further deepen mechanistic insights, citing more specific hormonal and metabolic pathways that underpin the atherogenic index’s role in prediabetes during menopause, in line with recent reviews.

**Response:** We thank the reviewer for this insightful suggestion. We have now deepened the mechanistic discussion by elaborating on the specific hormonal and metabolic pathways linking menopausal estrogen decline to dysregulated lipid/glucose metabolism.

“Beyond the well-known effects on insulin secretion and sensitivity(37, 38), recent advances highlight that estrogen deficiency impairs adipose tissue function, promoting a pro-inflammatory state and the release of free fatty acids, which further exacerbates insulin resistance and dysglycemia (39). Additionally, estrogen receptors in the pancreas and liver play direct roles in beta-cell survival and hepatic glucose output, the dysfunction of which post-menopause contributes significantly to metabolic dysregulation (40).”(Discussion: Paragraph 4)

**Reviewer #5:** 
**Comment 1:**Line 109: please present the number of subjects excluded due to missing data and outliers, which could potentially impact the interpretability and generalizability of the study results, particularly if a large number of subjects were excluded due to this reason.
**Response:**We thank the reviewer for this suggestion. As requested, we have now stated in the manuscript that 1,574 participants were excluded due to missing data and outliers.

“......and whose records contained outliers or missing values (n=1,574).” (Methods:Study design and participants)

**Comment 2:** Line 153-154: Please elaborate on the following statement “according to the critical value of RCS, AIP was dichotomized into low AIP and high AIP.” It is not clear to me what is the number of knots in the RCS model, whether the knot locations are pre-determined or data-driven, and how the cutoff is determined. These details are critical for understanding the conclusion of “approximately linear association between AIP and prediabetes” highlighted multiple times in the manuscript.

**Response:**We thank the reviewer for this important comment. The optimal number of knots was determined by comparing models with 3 to 5 knots using the Akaike Information Criterion (AIC), where a lower AIC indicates a better model-fit. The cutoff value of -0.24 for dichotomizing AIP was visually identified as the point on the fitted RCS curve where the risk of prediabetes began to increase. We have now elaborated on these details in the revised manuscript.

“A restricted cubic splines (RCS) model was also constructed to explore the dose-response relationship between AIP and prediabetes, and the number of knots was selected by comparing the Akaike Information Criterion.”(Methods:Statistical Analysis)

“We additionally utilized RCS with 4 knots to visualize the dose-response relationship between AIP and prediabetes.”(Results:Dose-response relationship)

**Comment 3:** For the provided “Joint analysis of AIP and menopausal status on prediabetes”, I have the following concerns:
**Comment 3-a:**If the cutoff of 0.24 for high-AIP and low-AIP is data-driven based on the RCS model, then it is questionable to conduct a stratified analysis based on AIP (high vs. low) and menopause status, since the data of AIP, prediabetes, and other covariates have been used twice in the modeling process.

**Response:**We thank the reviewer for raising this important methodological point, which we have carefully considered. We agree entirely that using a cutoff derived solely and directly from the present dataset for subsequent stratification could introduce bias due to data double-dipping.

However, we would like to respectfully clarify that the selection of the -0.24 cutoff was not purely data-driven from our RCS analysis. Instead, it was primarily informed and justified by existing clinical literature. For instance, the study by Yang et.al "Physical activity modifies the association between atherogenic index of plasma and prediabetes and diabetes: A cross-sectional analysis" Journal of Diabetes, 2024] has established and validated an AIP cutoff of -0.16 as a clinically significant threshold for predicting diabetes and prediabetes risks in other populations.

Therefore, while our RCS plot visually confirmed a risk increase around this previously established value, the decision to use -0.24 was based on prior external evidence rather than being optimized for our dataset. We believe this approach mitigates the concern of overfitting, as we are applying a pre-specified, clinically relevant cutoff to explore the potential interaction with menopausal status. The stratified analysis thus serves as an exploratory and hypothesis-generating step to assess whether the association between this established AIP risk category and prediabetes differs by menopausal status.

Notwithstanding, we appreciate the reviewer's caution and have added a note in the limitations section to explicitly state this context and recommend that future studies validate our findings using pre-defined, external cutoffs.

“Future multi-center, prospective, longitudinal studies using more objective assessment are needed to better clarify the relationships between AIP, menopausal status, and prediabetes across other ethnic and age groups. ”(Discussion: Paragraph 6)

**Comment 3-b:** The statement “the risk of prediabetes was significantly higher in the low AIP-postmenopausal (OR:1.12, 95%CI:0.91, 1.38)…” is not accurate. Based on Model 2, the OR between Low AIP-postmenopause vs. Low AIP-premenopause was 1.12 (0.91, 1.38), which was not statistically significant per the 0.05 alpha level.

**Response:**We thank the reviewer for correctly pointing out this inaccuracy. We have revised the relevant statement in the manuscript to accurately reflect that the association for the low AIP-postmenopausal group was not statistically significant (OR: 1.12, 95% CI: 0.91, 1.38), and we now emphasize that only the high AIP groups showed a significantly increased risk of prediabetes.

“Compared to the low AIP-premenopausal group, the risk of prediabetes was significantly higher in the high AIP-premenopausal (OR:1.51, 95%CI:1.24, 1.85) and high AIP-postmenopausal (OR:1.61, 95%CI:1.31, 1.98) groups.”(Results: Joint analysis of AIP and menopausal status on prediabetes)

**Comment 3-c:** The results reported in Table 3 does not clearly show that within each AIP strata, the menopause status significantly impact the risk of prediabetes (low AIP: 1.12 vs 1; High AIP: 1.61 vs. 1.51), even though the estimated ORs are numerically different. Therefore, the presented data do not provide strong support for the conclusion that “ the association between AIP and prediabetes varies according to menopausal status…)

**Response:**We thank the reviewer for this important comment. We agree that the data do not demonstrate a statistically significant effect modification by menopausal status. Accordingly, we have revised the discussion to temper our conclusions.

The text now clearly states that the formal interaction test was not significant and frames the findings as an observed "numerical pattern" rather than a definitive moderating effect.

“Furthermore, we explored the potential moderating role of menopausal status in the AIP-prediabetes association. It is important to note that a formal test for a multiplicative interaction was not statistically significant, suggesting that the strength of the association may not differ fundamentally between pre- and postmenopausal women. However, we observed a numerical pattern where the association was most pronounced in postmenopausal women with high AIP levels. This pattern is biologically plausible, as menopause is accompanied with the decline in ovarian function and changes in hormonal levels, which can affect lipid and glucose metabolism (35, 36).”(Discussion: Paragraph 4)

Yours sincerely,

Hongxia Zhuo

Institution and address: Hand Surgery Department, Union Hospital, Tongji Medical College, Huazhong University of Science and Technology, Wuhan, Hubei, P.R.China

Email: zhx2020zrj@163.com

Huiwu Han

Institution and address: Teaching and Research Section of Clinical Nursing, Xiangya Hospital of Central South University, Changsha, Hunan, P.R. China

Email: hanhw8888@csu.edu.cn
