# Supplementary material for: Does menopause influence the association between atherogenic index of plasma and prediabetes? A cross-sectional study in middle-aged Chinese women
Source: PLoS One. 2026 Feb 12;21(2):e0342644. doi: 10.1371/journal.pone.0342644 (PMC12900311; doi:10.1371/journal.pone.0342644)
Supplement: Attachment — Submitted filename: Response_to_Reviewers_auresp_2.docx [file pone.0342644.s009.docx]

Dear Editor and Reviewers:

We sincerely thank the reviewers and the editor for their further guidance and constructive feedback on our revised manuscript (ID: PONE-D-25-48687). We have carefully considered all additional comments and have made further revisions to address them. Our point-by-point responses are detailed below, with the revised text from the manuscript quoted in red.

**Reviewer #2:** This manuscript investigates an important and clinically relevant question using a large dataset and appropriate statistical methods.
However, several critical issues must be addressed :
**Comment 1:**The manuscript repeatedly implies that menopausal status modifies the association between AIP and prediabetes, despite a clearly non‑significant interaction term. The conclusions must be revised to reflect stratified associations rather than effect modification, and all overstated causal or moderating language should be removed.

**Response:** We sincerely thank the reviewer for this crucial methodological and interpretive insight. We fully agree that our original manuscript overstated the role of menopausal status as an effect modifier in the presence of a non-significant interaction term. We have comprehensively revised the manuscript to ensure all conclusions strictly align with our statistical findings, emphasizing **stratified and joint associations** rather than effect modification. The key revisions made in direct response to this comment are detailed below:

**(1) Clarification of the statistical finding：**

We have explicitly stated in the Methods, Results, and Discussion sections that **no statistically significant multiplicative interaction** between AIP and menopausal status was found (*P* = 0.973). This finding is now presented as a primary result, setting the correct context for subsequent interpretation.

**(2) Correction of terminology and interpretive language throughout the manuscript:**

**Abstract:** The objective was rephrased from “assess the potential role of menopause status” to “explore this association jointly with menopausal status.” The conclusion now states: “When considered jointly, a high AIP combined with postmenopausal status identified a subgroup with the greatest associated risk.”

**Introduction:** We now state that the study aimed to “explore the association between AIP and prediabetes among a sample of Chinese middle-aged women, and to assess the relationship jointly by menopausal status.” This revision removes any reference to a “moderating role” and accurately reflects the analysis performed.

**Discussion:** We have removed all claims of a “moderating effect”. The discussion now focuses on explaining the observed **risk pattern from the joint analysis** (i.e., “Therefore, while AIP is a significant risk factor irrespective of menopausal status, the co-occurrence of high AIP and postmenopause status identifies a subgroup of women at particularly elevated risk.”) from a biological perspective, without implying statistical interaction.

**(3) Summary of Changes:**

In essence, we have systematically replaced all language implying “effect modification” or “moderation” with accurate descriptions of our analytical approach (“joint analysis”) and findings (“joint association,” “risk in combined subgroups”). The narrative now consistently highlights that while AIP is a risk factor independent of menopausal status, the **combination of high AIP and postmenopausal status** identifies a subgroup of women at the highest risk, which is a clinically meaningful finding derived from joint analysis.

**Comment 2:**The Abstract, Discussion, and Conclusion require careful revision to ensure that all claims are strictly supported by the statistical evidence presented.

**Response:** We sincerely thank the reviewer for emphasizing the need for precise interpretation. We have carefully revised the Abstract, Discussion, and Conclusion throughout the manuscript to ensure all claims are strictly aligned with and supported by the presented statistical evidence. The key revisions are as follows:

Abstract: We have revised the conclusion to accurately reflect the findings from the joint analysis, removing any implication of effect modification. It now states: “When considered jointly, a high AIP combined with postmenopausal status identified a subgroup with the greatest associated risk.” This formulation is directly derived from the results of our joint analysis (Table 3).

Results Section (Interaction Analysis): The description of the interaction test has been rephrased to present it as an objective statistical assessment, avoiding any presupposition of a moderating effect. It now reads: “To test for a potential multiplicative interaction, an interaction term (AIP × menopausal status) was included in the fully adjusted logistic regression model (Model 2).”

Discussion: We have rewritten the relevant paragraph to base the discussion squarely on the statistical results. We first acknowledge the non-significant interaction test, then frame the subsequent discussion around the pattern observed in the joint analysis. The revised text begins: “Furthermore, we explored the association between AIP and prediabetes in relation to menopausal status... However, we observed a numerical pattern where the association was most pronounced in postmenopausal women with high AIP levels.” The biological plausibility discussed thereafter is now explicitly linked to explaining this observed risk pattern from the joint analysis, rather than an unsupported moderating effect.

Conclusion: The conclusion has been refined to focus on the practical implication of our joint analysis. It now suggests: “These findings underscore the potential of AIP as a simple, cost-effective biomarker for risk stratification in middle-aged women. The results suggest that evaluating AIP, especially in combination with menopausal status, could help identify women at the highest risk, enabling more targeted monitoring.”

In summary, we have ensured that the narrative from abstract to conclusion consistently distinguishes between the non-significant interaction test and the findings from joint analysis.

**Comment 3:**The current Data Availability Statement seems does not meet PLOS ONE requirements. The authors must either provide a publicly accessible dataset (or minimally sufficient anonymized data) or obtain explicit approval for a justified exception consistent with journal policy.

**Response:** We thank the reviewer for highlighting the importance of data availability. In accordance with PLOS ONE’s policy on minimal data sets, we have now deposited the de-identified analytical dataset comprising the 7,929 participants whose data were used for all statistical analyses and conclusions reported in the manuscript into the publicly accessible repository Mendeley Data (https://data.mendeley.com/datasets/7h7fh4k467/1). Additionally, we have updated the Data Availability Statement in the submission system to align with the journal’s required format, which now reads:

“All relevant data are available from the Mendeley Data repository at: https://data.mendeley.com/datasets/7h7fh4k467/1.”

This update ensures full compliance with PLOS ONE’s data-sharing policy.

**Comment 4:** A comprehensive language and formatting revision is necessary to correct grammatical errors, remove editing artifacts, and improve overall readability: There are instances of repeated words and careless typos (e.g., "Blood blood urea nitrogen" in Table 1). Please perform a careful proofread to remove these redundancies. And also variable names and terms are not capitalized consistently between the abstract, main text, and tables. Please apply a uniform style throughout.

**Response:** We thank the reviewer for highlighting these language and formatting issues. We have performed a thorough, line-by-line proofreading of the manuscript. Specifically, the instance of “Blood blood urea nitrogen” in the tracked-changes file was a display artifact resulting from overlapping revision marks. This has been corrected to “blood urea nitrogen” in the final, clean version of the manuscript (“Manuscript” file). All other grammatical errors, typos, and inconsistencies in terminology and expression have been uniformly corrected across the abstract, main text, and tables to ensure clarity and professional presentation. For example, we have standardized variable names and terms (e.g., “CVDs” has been revised to “CVD” throughout), removed abbreviations where appropriate (e.g., “GDM” and “IQR” are now spelled out in full), and refined phrasing in multiple instances, such as the opening sentence of the Introduction((e.g.,...but not meeting the diagnostic threshold for diabetes...), to improve overall fluency, accuracy, and consistency.

**Comment 5:** In summary, while the study has merit and potential, substantial revisions are still required to ensure methodological transparency, policy compliance, and accurate interpretation of findings.

**Response:** We sincerely thank the reviewer for the constructive summary and for acknowledging the merit of our study.We have undertaken substantial revisions to address the core requirements of methodological transparency, accurate interpretation, and policy compliance, as detailed in our specific responses to Comments 1–4. The key improvements are:

Methodological Transparency & Rigor: We replaced the initial data-driven AIP cutoff with the sample median for our primary analysis and added a pre-specified, literature-based sensitivity analysis, fully detailed in the Methods and Results.

Accurate Interpretation of Findings: We have revised the manuscript throughout to ensure all conclusions are strictly derived from the statistical evidence. Claims regarding menopausal status are now accurately framed around joint associations rather than unsupported effect modification.

Policy Compliance: In strict adherence to journal policy, the de-identified dataset underlying this study has been deposited in a public repository (Mendeley data.

Comprehensive Language and Formatting Review: A thorough proofreading was conducted to correct grammatical errors, typos, and inconsistencies, significantly improving the overall clarity and professionalism of the manuscript.

We believe these revisions have significantly strengthened the validity and clarity of our work, and we are grateful for the reviewer’s insights which have greatly improved the manuscript.

**Reviewer #5:**

**Comment 1:** I would like to thank the authors for the additional clinical background and literature regarding the selection of -0.24 AIP cutoff, while I remain unconvinced by the authors’ response regarding the double-dipping concern. The authors state that the –0.24 cutoff was clinically informed and therefore pre-specified. However, as acknowledged in their earlier response to my comment #2, the cutoff was “visually identified” from the RCS curve using the study data. This indicates that the threshold was derived post hoc, not determined a priori, and therefore cannot be considered pre-specified.
 The subsequent reuse of the same dataset to evaluate the interaction between AIP and menopausal status on prediabetes then constitutes double dipping, which undermines the validity and credibility of the resulting estimates. Notably, the fully adjusted logistic regression model shows no meaningful interaction (OR = 1.00, 95% CI: 0.83–1.22), whereas the results produced through this data-driven cutoff suggest otherwise (Table 3), further underscoring the methodological concern.
 To avoid this issue, the authors could consider using a threshold established in prior literature (e.g., –0.16) or a distribution-based cutoff such as the median or a relevant quantile. These alternatives, while not strictly pre-specified, are not derived from the outcome data and therefore do not raise the same risks of double dipping.

**Response:**We sincerely thank the reviewer for raising this critical methodological point. We fully agree that using a data-driven cutoff visually identified from an outcome-associated RCS curve for subsequent hypothesis testing constitutes a form of “double-dipping” and can invalidate the statistical inference.

In direct response to this concern, we have revised our primary analysis to eliminate this bias and have performed an additional sensitivity analysis to ensure the robustness of our conclusions. The revisions are as follows:

(1) Revised Primary Analysis (Median-Based Cutoff):

We have changed the method for dichotomizing the AIP in our main joint analysis. Instead of the point visually identified from the RCS curve, we now use the sample median of AIP as the cutoff point. The median value in our study cohort is -0.24. This approach defines the exposure category based solely on the sample distribution, independent of the outcome variable, thereby completely avoiding the “double-dipping” issue.

We have updated the Methods section accordingly: “For the primary joint analysis, AIP was dichotomized into low and high groups based on the sample median value (-0.24). To test the robustness of our findings, we performed a sensitivity analysis in which AIP was dichotomized using a cut-off of -0.16, which has been validated in prior research (23)..” (Methods:Statistical analysis)

An important observation is that the sample median (-0.24) is numerically identical to the inflection point noted in our initial exploratory RCS curve. Therefore, while the methodological justification has been fundamentally strengthened, the dichotomization threshold and all numerical results presented in Table 3 remain unchanged. The estimates for the joint associations are preserved but are now methodologically robust.

(2) New Sensitivity Analysis (Literature-Informed Cutoff):

To further address the reviewer’s suggestion and demonstrate that our findings are not contingent on a specific cutoff choice, we conducted a sensitivity analysis using an alternative, clinically-informed AIP cutoff of -0.16, as referenced in prior literature. This cutoff was not derived from our dataset. The results of this analysis are presented in Table S5 (S6 Apeendix). As reported in the revised Results section, the pattern of association from this sensitivity analysis is fully consistent with our primary findings from Table 3.

“In sensitivity analysis using an AIP cut-off value of -0.16 (Table S5, S6 Appendix), the pattern of association remained consistent.” (Results:Joint analysis of AIP and menopausal status on prediabetes)

We believe these revisions directly and satisfactorily resolve the methodological issue raised, significantly strengthening the validity and credibility of our findings. We thank the reviewer for this insightful comment, which has improved the rigor of our work.

Yours sincerely,

Hongxia Zhuo

Institution and address: Hand Surgery Department, Union Hospital, Tongji Medical College, Huazhong University of Science and Technology, Wuhan, Hubei, P.R.China

Email: zhx2020zrj@163.com

Huiwu Han

Institution and address: Teaching and Research Section of Clinical Nursing, Xiangya Hospital of Central South University, Changsha, Hunan, P.R. China

Email: hanhw8888@csu.edu.cn
